# COVID‐19 and Inequalities*

**DOI:** 10.1111/1475-5890.12232

**Published:** 2020-07-14

**Authors:** Richard Blundell, Monica Costa Dias, Robert Joyce, Xiaowei Xu

**Affiliations:** ^1^ University College London; Institute for Fiscal Studies; ^2^ Institute for Fiscal Studies; University of Porto; ^3^ Institute for Fiscal Studies; ^4^ Institute for Fiscal Studies

**Keywords:** inequality, employment, health, ethnicity, COVID‐19

## Abstract

This paper brings together evidence from various data sources and the most recent studies to describe what we know so far about the impacts of the COVID‐19 crisis on inequalities across several key domains of life, including employment and ability to earn, family life and health. We show how these new fissures interact with existing inequalities along various key dimensions, including socio‐economic status, education, age, gender, ethnicity and geography. We find that the deep underlying inequalities and policy challenges that we already had are crucial in understanding the complex impacts of the pandemic itself and our response to it, and that the crisis does in itself have the potential to exacerbate some of these pre‐existing inequalities fairly directly. Moreover, it seems likely that the current crisis will leave legacies that will impact inequalities in the long term. These possibilities are not all disequalising, but many are.

## I. Introduction

Much of the debate about the impacts of the COVID‐19 pandemic, our responses to it, and the longer‐term legacy that it will leave has quickly become a discussion about various forms of inequality. In the UK, the years leading up to the COVID‐19 crisis had left many households in a precarious position. A lack of pay growth at the bottom of the distribution of household earnings meant the finances of many households were under strain prior to the crisis, and years of austerity reduced the scope of the state as an insurer against future shocks. The economic shock associated with COVID‐19, which resulted from the lockdown and severe reduction in economic activity of many sectors of the economy, will not affect all in the same way. Indeed, it is becoming clear that it will interact with many of the pre‐existing inequalities along dimensions such as gender, ethnicity, age and geography. Moreover, this pandemic‐induced crisis is having impacts across multiple related aspects of life, from health to jobs and to family life, and these impacts are interrelated. The most vulnerable groups by socio‐economic background and health status are also those that may be hit the hardest.

In this paper, we bring together what has emerged so far about the impacts of the crisis on inequalities across several key domains of life, including on employment and ability to work, the division of work and domestic responsibilities in families with children, education investments and health. We relate these to existing dimensions of inequalities, including socio‐economic background, education, gender, ethnicity and geography. In doing so, we make a few overarching points. First, the deep underlying inequalities and policy challenges that preceded this crisis, including those that developed since the last major economic crisis in the late 2000s, are crucial in understanding the complex impacts of the pandemic itself and our response to it. This crisis is highlighting the need to tackle some of these disparities and the initial policy responses aimed precisely at protecting those who came into this crisis in precarious circumstances. Second, the crisis does in itself have the potential to exacerbate some inequalities fairly directly – including, for example, the economic disadvantages faced by the young. Third, it seems certain that this episode will leave longer‐term legacies that will impact on inequalities, and the policies that might best tackle them, in years to come.

We find that younger workers, those on low incomes and the self‐employed are more likely to have lost their job or experienced a drop in economic activity that is likely to result in a reduction in earnings during the lockdown. Young workers, particularly those who would have entered work this year, face potentially long‐lasting scarring from the collapsing labour market. The jobs of key workers – who are more likely to be lower paid, female and from some ethnic minority backgrounds – are less likely to be at risk but they often face more health risks. Health impacts have been unequal and have been more serious among workers in certain occupations that are more exposed to social contact, among ethnic minority groups and in poorer localities. Parents are coping with huge additional demands on their time to care for and educate their children from home, and poorer families have been receiving less support from schools in doing so. In contrast, parents in better‐off families and with higher levels of education are more likely to be able to carry out their work activities from their home, to have space at home to educate their children, and to have savings to cover unforeseen expenditures.

It is therefore becoming increasingly clear that this crisis will leave many challenging legacies for inequality. These might be compounded by other major effects of this crisis – for instance, on the market power of big firms if smaller firms struggle to survive, or on the capacity of the government to address these inequalities given the record peacetime levels of debt. But the crisis may bring opportunities too. An increase in remote working could be especially helpful for mothers’ careers, and the longer hours that fathers are spending with their children during this period might help to accelerate changes in gender norms. Widespread working from home may reduce the dominance of London. And we might see changes in attitudes towards the welfare system and social insurance and towards key workers, and more salience given to inequalities such as those between ethnic groups. Policymakers have rightly been consumed by the immediate response to the crisis, but attention should already be turning to the longer‐term effects. In this paper, we discuss some of the potential long‐lasting consequences from this crisis.

The rest of the paper is organised as follows. We set the scene by overviewing some key dimensions of existing inequalities in Section II. Section III examines how the policies enacted to counter the spread of the virus, and the virus itself, interact with existing patterns of inequality. It looks into who is most affected by sector shutdowns, which workers can continue working from home, how families deal with the additional burden of caring for their children from home, what resources they have available to support the education of their children and how the health risks of COVID‐19 are distributed. Section IV concludes with a discussion of what our findings imply for future inequalities.

## II. Inequalities before the pandemic

Inequalities existed along many dimensions before the pandemic hit, across the population and between different groups – by gender, ethnicity, age and geography. In the UK, hourly wages of women are a fifth lower on average than those of men, and progress towards closing the gender wage gap had stalled in the last decade.^1^ There are substantial differences by ethnicity, both between and within genders: employment rates among white British individuals are 73 per cent for women and 80 per cent for men, but among Pakistanis and Bangladeshis they are 39 per cent and 75 per cent respectively.^2^ The Great Recession that began around 12 years ago had hit the pay and employment of young adults the hardest, and likely contributed to the acceleration of a trend of widening economic inequalities between generations. Plummeting homeownership among young people meant that young people today have significantly less wealth than previous generations had at the same stage in life.^3^

**FIGURE 1:**
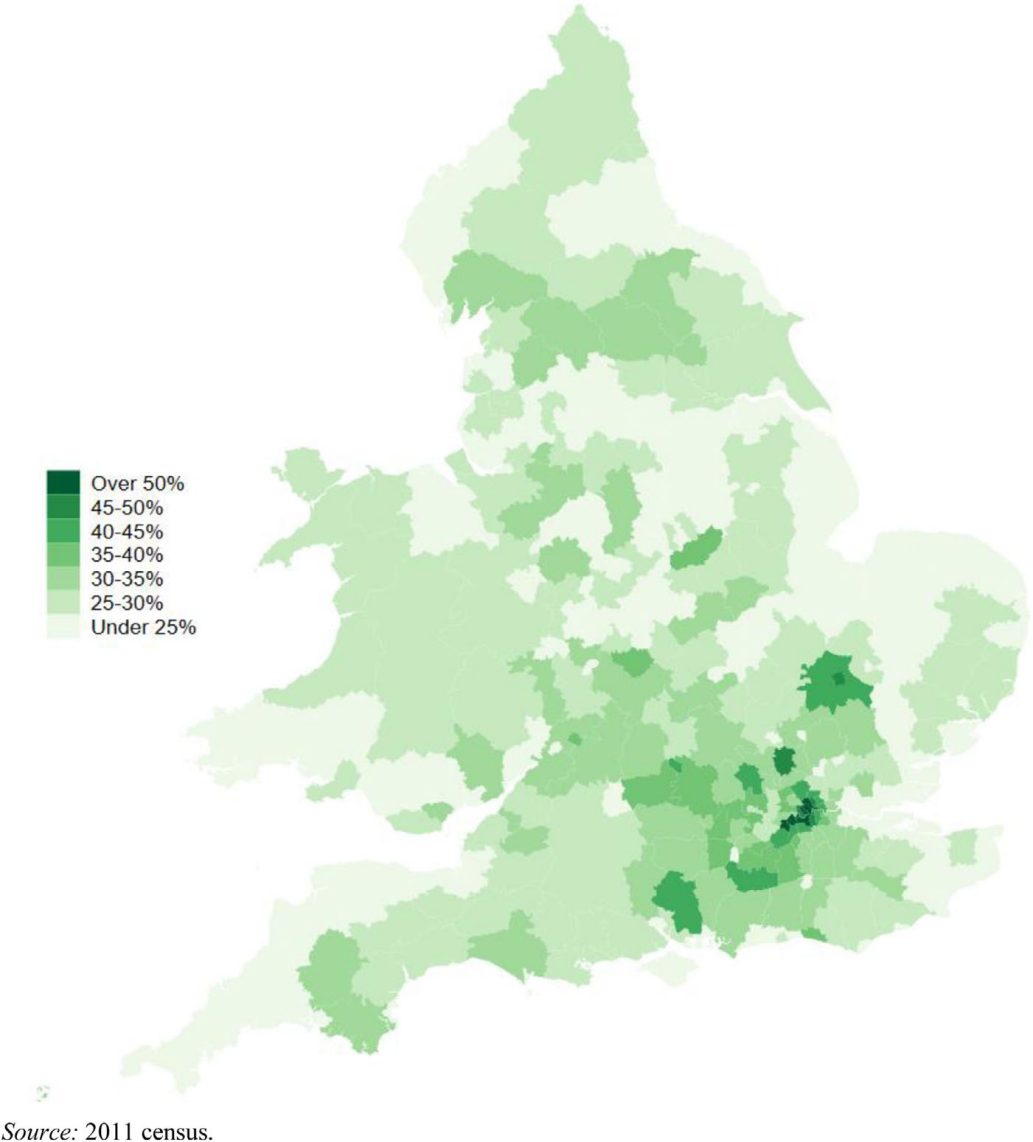
Share of population with post‐A‐level qualifications

Geographical inequalities in the UK are large and persistent. Over the past few decades, London has pulled away from the rest of the country. Average weekly earnings among full‐time employees are a third higher than the UK average – though high housing costs mean that Londoners’ real living standards are not as high as income differentials suggest.^4^ Disparities in economic outcomes closely mirror educational disparities across the country and the growing concentration of skilled workers in the capital.^5^ Figure 1 shows that whilst in vast swathes of the country less than a quarter of the population have post‐A‐level qualifications, in central London the majority of people have higher qualifications. Spatial inequalities exist in health as well as incomes. Men born in the 10 per cent most affluent areas could expect to live nearly 10 years longer than those born in the most deprived areas, and women nearly 8 years longer, and these gaps have widened over time.^6^

Income inequality in the UK, as measured by the Gini coefficient, was high by international standards before the global financial crisis, having risen steeply during the 1980s.^7^ Inequality in household labour incomes continued to rise between the mid 1990s and the Great Recession. But households across the distribution did at least see some real increase in their earnings over this period, and large increases in cash transfers – especially through higher in‐work benefits, and in particular the expansion of tax credits – propped up incomes towards the bottom.

**FIGURE 2:**
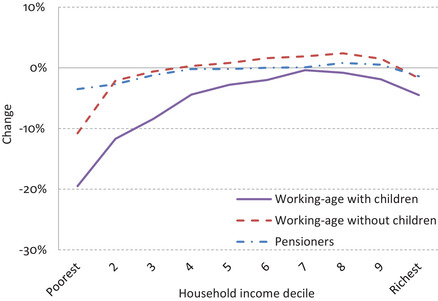
Change in net household incomes due to tax and benefit reforms, by income, 2010 to 2019 *Note*: Shows long‐run effect of tax and benefit changes with all reforms fully rolled out. Household incomes calculated after taxes and benefits. Deciles based on net household incomes equivalised using modified OECD equivalence scale. *Source*: Family Resources Survey 2017–18 and the IFS tax and benefit microsimulation model, TAXBEN.

In contrast, the decade immediately before the onset of COVID‐19 was itself largely a hangover from the Great Recession, characterised by stagnating real earnings, large cuts to the welfare system and an increase in ‘solo’ self‐employment. Short‐lived signs of a pick‐up in real earnings growth in the mid 2010s were choked off by a rise in inflation associated with the depreciation of sterling in the wake of the Brexit referendum of 2016 – an increase in inflation which also further eroded the value of working‐age benefits, since the government had already committed to freeze them as part of its ongoing austerity measures. Since the Great Recession, real earnings barely increased anywhere in the income distribution, and – unlike the pre‐2008 period – large increases in cash transfers that had previously been helping to compensate for this were no longer doing so. Figure 2 looks at the set of benefit cuts made since the Great Recession and shows the large impact of these on incomes at the bottom of the working‐age distribution, especially for households with children.

One common feature of a number of the cuts to benefits made since 2010 is that they have weakened the link between benefit entitlements and the actual costs that families face. That is obviously true of the tendency for working‐age benefits not to be increased in line with inflation between 2013 and 2020, but it is also true of a number of more specific examples: the introduction of the overall benefits cap (which capped the total value of benefit entitlements, meaning, for example, that support for rents was effectively curtailed if you also claimed large amounts of support for children, and vice versa), the two‐child limit in tax credits and universal credit (which limits payments to the first two children in the family), the so‐called ‘bedroom tax’ (which reduces housing benefit entitlements below actual rent levels for social renters who are deemed to have bedrooms that they do not need) and changes to the local housing allowance for private renters (which linked entitlements to rents in 2012, rather than the present).

The UK welfare system, unlike those in many other European countries, already has very little emphasis on earnings replacement for those who lose employment – welfare entitlements bear almost no relation to previous earnings. These cuts further weakened the extent to which the state would help people meet their spending commitments in the event of an unexpected income shock. More generally, over a long period stretching back to the 1980s, the value of out‐of‐work benefits has drifted further and further behind the earnings levels of those in work. The relative focus of the system has shifted significantly towards income top‐ups for those in work but on low earnings. A consequence is that the system has become more focused on insuring against falls in earnings and less focused on insuring against falls in employment.^8^

All these choices are coming into sharp focus during the COVID‐19 crisis, where many families who probably never expected to need the welfare system so much will find just how little insurance it can provide. Adam, Miller and Waters (2020) estimate that on the eve of the COVID‐19 pandemic, 16 per cent of employees would have lost more than 80 per cent of their family income if they were made unemployed, and a further 20 per cent would have lost between 60 and 80 per cent of their income.

Coming into this crisis, a large fraction of people had very little savings to tide them over in times of crisis and many had high levels of debt. Sturrock (2020) shows that in 2017–18, nearly a third of individuals towards the bottom of the household income distribution, and around a fifth of those in middle‐income households, said that they would be unable to manage a month if their household lost its main source of income. A substantial share of people had very high levels of debt prior to the pandemic, with around one in ten people in low‐ and middle‐income households spending more than a fifth of their net income repaying consumer debt. Low savings and high debt meant that many households were unable to privately insure against income shocks and were reliant on insurance provided by the state.

In summary, the years leading up to the COVID‐19 crisis – and in particular the long hangover from the last economic crisis of the late 2000s – had left many households in a precarious position. This is both because their own finances had already been under strain and because the role of the state as an insurer against possible future shocks had been further cut back. As a result, the UK government had to move very quickly to set up temporary employment insurance schemes from scratch when the pandemic hit. These policies, whilst generous – covering 80 per cent of lost earnings of furloughed and self‐employed workers up to £2,500 a month – still left many individuals falling through the cracks^9^ and created a sharp inequality between workers who were furloughed and those who were laid off and therefore reliant on the pre‐existing benefit system. As the furlough and self‐employment schemes are gradually unwound over the coming months, the low levels of insurance provided by state benefits will once again be drawn into sharp focus.

## III. How does the pandemic interact with existing inequalities?

Since the onset of the pandemic, the public health response in the UK, as in many other countries, has involved a sweep of changes that have brought existing inequalities into sharper focus. These include the mandated shutdown of entire sectors such as hospitality and retail (excluding food and pharmaceuticals), school and nursery closures, and widespread working from home. The economic impacts of these changes have not been felt equally across the population, but have interacted with existing divides by income, age, gender and ethnicity and in many cases exacerbated existing inequalities. The health impacts of the virus have also not been evenly spread so far, with higher death rates among certain occupations, ethnic minority groups and poorer localities. This section examines how the policies enacted to counter the spread of the virus, and the virus itself, interact with existing patterns of inequality.

### 1. Sector shutdowns

Entire sections of the economy were ordered to shut down by the government to prevent the virus from spreading. In the UK, as in many other countries, these included all non‐food, non‐pharmaceutical retail, hotels and restaurants, and arts and leisure services. Passenger transport was also greatly reduced due to stay‐at‐home orders, and air travel has largely been suspended.

**FIGURE 3:**
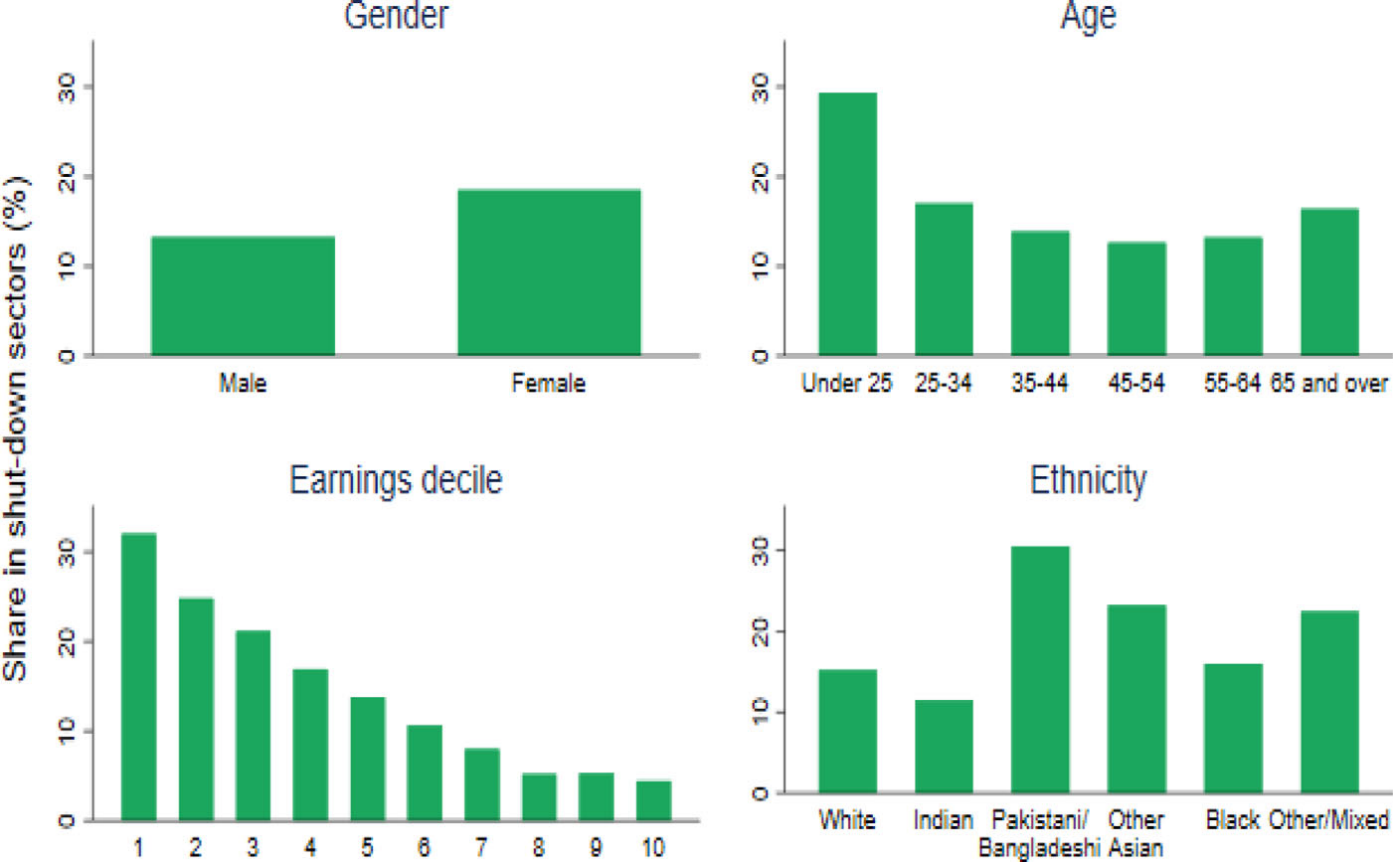
Share of workers in shut‐down sectors pre‐crisis, by socio‐demographic and socio‐economic group *Note*: Breakdown by earnings refers to employees only. Excludes those in full‐time education. Shut‐down sectors include (with four‐digit Standard Industrial Classification (SIC) codes in parentheses): non‐food, non‐pharmaceutical, non‐internet retail (4719, 4730–4772, 4776–4789); passenger transport (4910, 4931–4939, 5010, 5030, 5110); accommodation and food (5510–5630); travel (7911–7990); childcare (8510, 8891); arts and leisure (9001–9329 except ‘artistic creation’ 9003); personal care (9601–9609 except ‘funeral and related activities’ 9603); and domestic services (9700). *Source*: Labour Force Survey, quarters 1–4 2019, waves 1 and 5 only.

The direct and immediate economic impact of the lockdown has therefore been concentrated among workers employed in these shut‐down sectors. As Figure 3 shows, these workers are disproportionately female, young and low‐paid. We will see that mothers are more likely to have quit or lost their job, or to have been furloughed, since the start of the lockdown. Workers under the age of 25 are twice as likely to work in a shut‐down sector as those aged 25 and over, whilst employees in the bottom 10 per cent of the weekly earnings distribution are seven times more likely than those in the top 10 per cent to do so. Certain ethnic minority groups, in particular Pakistani and Bangladeshi workers, are heavily concentrated in the passenger transport (taxi driving) and food and beverage sectors, and are likely to be hardest hit by the lockdown – especially since they are relatively likely to be the sole earners in their households.^10^

Data collected since the start of the pandemic confirm what we would expect given the patterns documented using pre‐crisis data above. Based on a survey of nearly 4,000 individuals conducted at the end of March, Adams‐Prassl et al. (2020) find that younger workers and those on low incomes are much more likely to have lost their job due to COVID‐19, and are more likely to have experienced a reduction in earnings, than older and higher‐income workers. They were also more likely to expect larger further cuts to their incomes. The authors’ findings also support the expectation we might have about whose livelihoods are most precarious in a situation like this, with the self‐employed and workers in less secure work arrangements (for example, zero‐hours contracts) being more likely to report being negatively affected. A larger study of over 17,400 respondents from the Understanding Society panel also found that the fall in employment was greatest among the young and those with lower levels of education, whilst the self‐employed and those on zero‐hours contracts experienced the largest fall in hours worked.^11^

The mandated closure of a large number of sectors also has implications for households’ spending. Here, differences in spending patterns prior to the pandemic mean that not all households will be equally affected. A recent study by Crawford et al. (2020) shows that before the onset of COVID‐19, households in the top quintile of the income distribution spent nearly a third of their total expenditure on services that have been affected by the shutdown: transportation and ‘hospitality and leisure’, a category that includes holidays, hotels, restaurants, personal care (including hairdressing) and culture (including museums and theatres). Given that these services have either been forbidden or hugely scaled back, higher‐income households who have not suffered income falls may effectively find themselves fairly automatically saving significant amounts of money while lockdowns or substantial forms of social distancing persist. On the other hand, as documented above, the lack of consumer demand in these sectors is what is now removing the livelihoods of many lower earners. Meanwhile, low‐income households, who are likely to suffer the biggest hit to earnings, will find it difficult to scale back their expenditure: prior to the pandemic, over half of their spending was on necessities such as groceries, housing and utilities that are difficult to adjust.

### 2. Working from home and key workers

Given the nature of this pandemic, the ability to work from home (and whether workers feel safe in doing their work, without conflict with their employer) is likely to be a key determinant of whether workers stay actively engaged in work whilst social distancing measures remain in place. Even in sectors that are not under lockdown, the measures to contain the spread of the disease and incentivise work from home made many workers temporarily or permanently redundant. There are good reasons to expect that these workers will be disproportionately concentrated in occupations that support the main production activities and must be carried out in the workplace, such as cleaning or security services.

**FIGURE 4:**
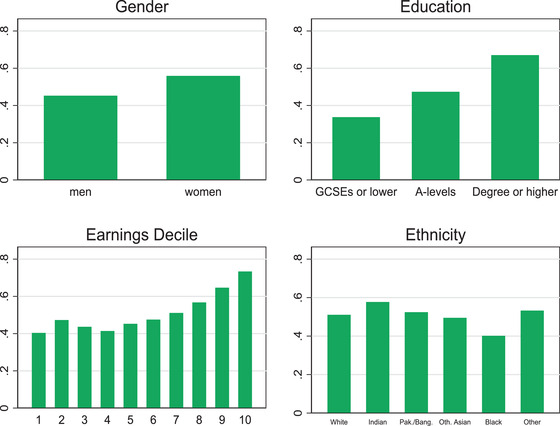
Share of workers in sectors not in lockdown who can work from home, by socio‐demographic and socio‐economic group *Note*: O*NET data used to identify occupations that are amenable to working from home, using classification in Dingel and Neiman (2020). ‘Pak./Bang.’ stands for Pakistani or Bangladeshi. ‘Oth. Asian’ are Asian ethnicities other than Indian, Pakistani or Bangladeshi. ‘Other’ are mostly mixed raced ethnic backgrounds. *Source*: Labour Force Survey, quarters 1–4 2019, waves 1 and 5 only.

**FIGURE 5:**
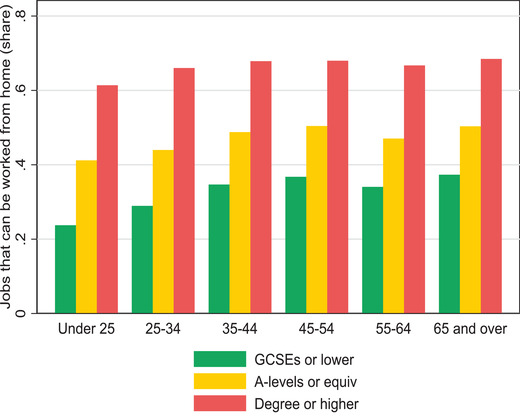
Share of workers in sectors not in lockdown who can work from home, by age and education *Note*: O*NET data used to identify occupations that are amenable to working from home, using classification in Dingel and Neiman (2020). *Source*: Labour Force Survey, quarters 1–4 2019, waves 1 and 5 only.

Figure 4 shows how the ability to work from home is distributed among workers in sectors that are not in lockdown, by socio‐demographic and socio‐economic groups. Clearly, it is those with higher levels of education and higher earnings who are more likely to be able to carry out their work activities from their home. Furthermore, Figure 5 shows that education is likely to be especially protective of the jobs of younger workers. While those under the age of 25 are more exposed to jobs that cannot be worked from home even if their sectors are not in lockdown, the differences across age groups are larger for those with GCSE qualifications or less. These differences across education levels and cohorts accentuate the hit taken by low‐paid workers, who are also disproportionately concentrated in lockdown sectors, as shown in the previous subsection. We also find that men and black workers concentrate in occupations that are less amenable to home working, which can partly counterbalance their lower concentration in lockdown sectors.

While many workers were essentially forced to stop work, ‘key workers’ – including workers in health and social care, security and some wholesale and retail – were conversely urged to continue working despite the dangers posed by COVID‐19. While on the one hand this meant that their livelihoods were not put in jeopardy in the same way as for many other workers, given the nature of their jobs they can seldom carry them out from their home and may therefore be at particularly high risk of infection. Rates of COVID‐19 deaths among men were between 2 and 3.7 times higher in a number of key worker occupations – specifically care workers, taxi drivers, bus and coach drivers, and sales and retail assistants – than among the general population.^12^ Data as at 5 June from random swab tests show that infection rates remain far higher among health and social care workers than among others.^13^

**FIGURE 6:**
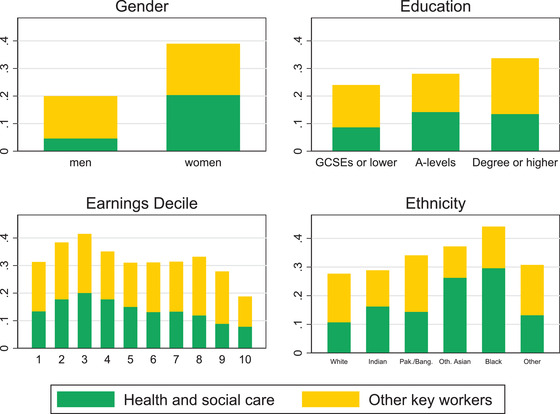
Share of key workers among individuals employed in sectors not in lockdown, by socio‐demographic and socio‐economic group *Note*: ‘Pak./Bang.’ stands for Pakistani or Bangladeshi. ‘Oth. Asian’ are Asian ethnicities other than Indian, Pakistani or Bangladeshi. ‘Other’ are mostly mixed raced ethnic backgrounds. *Source*: Labour Force Survey, quarters 1–4 2019, waves 1 and 5 only.

Figure 6 shows how key workers are distributed across socio‐economic groups. They are more evenly spread across earnings deciles than workers in shut‐down sectors or those who can work from home. Workers in health and social care are somewhat more concentrated in the bottom half of the income distribution, although the even spread across education groups reflects the variety of skills used in this sector. Where differences are very pronounced is across gender and ethnicity. Women not working in lockdown sectors are twice as likely as men to be key workers, and over four times as likely to work for the health and social care sector. This concentration of female workers in key sectors, particularly in health and social care, may induce some families to prioritise the work of the woman over the man during this period. The choices of who will work and who takes on other responsibilities will be especially important for families with young children, who are now facing huge pressures to replace in‐house the care that was formerly provided in schools and childcare facilities. We discuss these issues and their potential long‐term consequences below.

**FIGURE 7:**
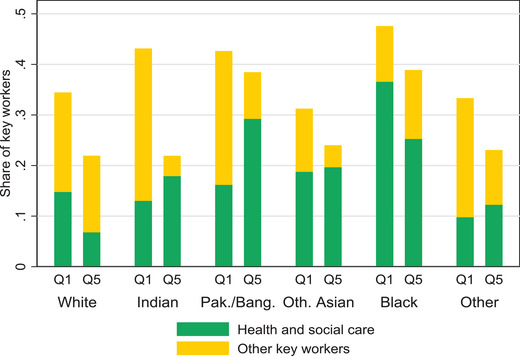
Share of key workers among individuals employed in sectors not in lockdown, by ethnicity and earnings quintile *Note*: ‘Pak./Bang.’ stands for Pakistani or Bangladeshi. ‘Oth. Asian’ are Asian ethnicities other than Indian, Pakistani or Bangladeshi. ‘Other’ are mostly mixed raced ethnic backgrounds. *Source*: Labour Force Survey, quarters 1–4 2019, waves 1 and 5 only.

Figure 6 also shows that key workers are disproportionately represented among ethnic minorities, particularly of black backgrounds. Recent work has highlighted the especially high occupational risks that the black community is exposed to during this pandemic, and how these are likely to be a determinant of the high infection and mortality rates among them.^14^ The differences across ethnicity and income groups are larger for the health and social care sector than for other key services. Moreover, the strong preponderance of key workers in the health and social care sector among black people – as compared with those from other ethnicities – spans across economic groups. Figure 7 shows this, by decomposing the distribution of key workers by ethnicity and economic status, contrasting the top and bottom fifths (Q5 and Q1) of the earnings distribution. The figure also reveals that key workers are disproportionately concentrated in the bottom group. The differences across earnings groups are larger among those of white and Indian ethnicities and tend to be larger for those working in sectors other than health and social care (although this is not the case for all ethnicities).

**FIGURE 8:**
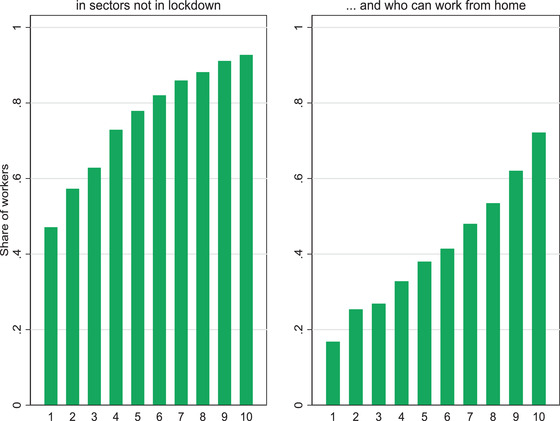
Share of workers in sectors not in lockdown and who can work from home, excluding key workers, by decile of earnings distribution *Note*: O*NET data used to identify occupations that are amenable to working from home, using classification in Dingel and Neiman (2020). *Source*: Labour Force Survey, quarters 1–4 2019, waves 1 and 5 only.

Figure 8 brings together the sector and occupation information to investigate how the ability to continue to work in a safe environment varies over the income distribution. We exclude key workers, who we know are especially exposed to contagion but whose livelihoods have not been otherwise compromised by this crisis. The figure highlights huge inequalities. It shows, in the left‐hand panel, that less than half of the workers in the bottom tenth of the earnings distribution pre‐crisis are employed in sectors that have not been shut down. In contrast, workers in the top earnings group are twice as likely to be employed in these sectors. The ability to continue working from home is also extremely unevenly distributed. The right‐hand panel of Figure 8 shows that fewer than one in five non‐key workers in the bottom earnings group have jobs that can be done from home in sectors that remain active. That is only one‐third of the workers in that earnings group who are employed by sectors not in lockdown. But among non‐key workers in the top income group, three in four have jobs that are amenable to home‐based work in sectors that remain active.

The figures we have discussed so far show that the ability to continue working through the lockdown period, and to work safely, is distributed very unevenly by gender, ethnicity, education and earnings. These were all key dimensions of inequality before the crisis; the heterogeneous effects of the crisis on the ability to work and earn along these dimensions may have made these inequalities even more pronounced. We now look into how inequalities may also be building up in families, in how responsibilities are shared between spouses and in access to education activities among children.

### 3. Families with children

With the closure of schools, nurseries and other childcare facilities for all but the children of essential workers and vulnerable children, parents were typically left with sole responsibility for caring for their children, and with much greater responsibility for their education as schools made the shift to online learning. Against a backdrop where female employment rates were at record highs, this has created unprecedented demands on the time of parents: 21^st^ century maternal employment rates have suddenly been coupled with the almost‐complete removal of the childcare facilities that had made those employment rates possible. Doing market work without the option of outsourcing childcare to the market at the same time will be particularly difficult for those who cannot work from home and who have no access to informal forms of childcare (which is now especially likely, given the restrictions on mixing with other households).

**FIGURE 9:**
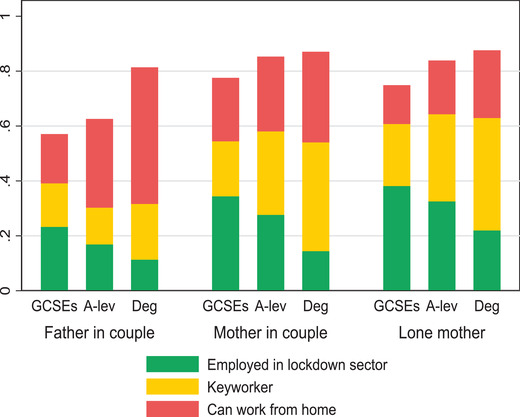
Job types of working mothers and fathers before the crisis, by family arrangements and education *Note*: O*NET data used to identify occupations that are amenable to working from home, using classification in Dingel and Neiman (2020). The education levels match those in the previous subsection: ‘GCSEs’ includes those leaving education at the age of 16 with minimal or no qualifications; ‘A‐lev’ are those with qualifications acquired at the end of high school or equivalent; and ‘Deg’ are those with university‐level qualifications in programmes that require at least three years for completion. *Source*: Labour Force Survey, quarters 1–4 2019, waves 1 and 5 only.

Figure 9 shows how vulnerable the working arrangements of mothers and fathers were to the shock of COVID‐19 and subsequent lockdown of parts of the economy. It is well known that many more mothers than fathers were not working before the crisis, particularly among workers with the lowest levels of formal education and single mothers (for whom employment rates were as low as 53 per cent among those who left school with only GCSE qualifications or less). Some of these mothers may have been preparing to return to work, and this was made more difficult by the crisis. But the large majority of them are likely to be going through long‐term work interruptions related to childcare demands that are unaffected by this crisis.

There are large differences across gender and education in the ability of parents who were working before the crisis to continue to do so, and to do so from home – where it is both safer and easier to combine work with the additional childcare responsibilities that parents have to shoulder. Mothers, particularly lone mothers, were more likely to work for sectors that have been shut down. Overall, almost 40 per cent of working single mothers with the lowest level of qualifications were employed in lockdown sectors prior to the crisis. That figure compares with 34 per cent and 23 per cent of similarly educated mothers and fathers in couples, respectively. Working mothers are also more likely than fathers to be key workers, particularly among those with the highest level of qualifications, but the differences are nearly offset by mothers being otherwise less likely to be able to continue working from home. These two features are expected to be key in facilitating the ability of parents to continue working during the lockdown. They are much more prevalent among educated parents. For instance, only one in three working fathers of the lowest education group are either key workers or can continue working from home; that figure increases to 70 per cent of working fathers in the top education group. So lower‐educated parents are not only more likely to work in sectors that have been shut down; in addition, if their previous job remains open, they are less likely to be able to do it from home and so are more likely to struggle to make it compatible with their additional childcare responsibilities.

There are reasons this crisis will affect mothers and fathers differently in addition to what happens to their job. If, as has happened traditionally, mothers take a disproportionate share of the additional childcare and housework responsibilities, their ability to do paid work and to keep the levels of productivity they had pre‐crisis will be limited even if their jobs remain active. To investigate these issues, researchers from the Institute for Fiscal Studies (IFS) and the Institute of Education (IoE), with funding from the Nuffield Foundation, have collected data for over 3,500 opposite‐gender couples with children aged 4–15, recording demographic and economic information, as well as how parents and their children were spending their time under lockdown during the first two weeks of May. In order to keep the survey a manageable length for families, it asked about time use in one‐hour slots and allowed for more than one activity to be reported in each slot. Compared with the most detailed time‐use surveys, such as the 2015 UK Time Use Survey which records activities in finer 10‐minute intervals, these data do not measure precisely how long respondents spent on a particular activity. Instead, they are better suited to reveal the sequence and relative predominance of activities carried out through the day.

Recent work by Andrew et al. (2020a) uses this study to examine inequalities in working status and the time use of couples with children aged 4–15. Their findings are in line with recent studies arguing that this crisis, contrary to previous recessions, is exposing mothers more than fathers to the risk of job loss due to either their jobs being interrupted or to their incapacity, as mothers, to combine huge increases in domestic responsibilities with the demands from paid work.^15^ Comparing mothers and fathers who were working for pay in February, Andrew et al. find that it is mothers who are more likely to have lost their jobs, either temporarily (34 per cent and 30 per cent for mothers and fathers, respectively) or permanently (16 per cent versus 11 per cent). However, recent estimates based on the first COVID‐19 wave of the UK Household Longitudinal Study^16^ suggest that the effect of the crisis on the working status of working‐age women and men (not only parents) was more evenly distributed than what Andrew et al. found for parents. Overall, there are offsetting factors at play when it comes to gender differences in the impact of this crisis on employment: for example, women are more likely to be in shut‐down sectors but also more likely to be key workers or in jobs that can be done from home. More data that will soon be available will be important in shedding further light on how all those effects are playing out.

Andrew et al. (2020a) also find important differences in the effects of lockdown on the working status of parents from different education groups. In particular, both mothers and fathers who left school with little or no qualifications are 15 percentage points more likely to have temporarily or permanently stopped working than those with a university degree. Moreover, working from home is much more common among the more educated parents. More than 50 per cent of parents in the lowest education group who continue to work for pay do so outside their home; that proportion drops to 25 per cent for mothers and 19 per cent for fathers in the university‐educated group.

Changes in employment are not the only way in which the lockdown is affecting families and how mothers and fathers allocate their time. The large increase in childcare and education activities that need to be carried out at home means that parents are now facing huge additional demands for their time. In their study, Andrew et al. (2020a) show how mothers and fathers are spending their time in domestic and professional responsibilities, depending on their work status. They show, not entirely unexpectedly, that mothers are always doing more childcare and housework on average than fathers, regardless of their working status. In turn, mothers who work for pay do fewer hours in paid work than fathers do, but the difference does not offset gaps in childcare and housework time. But the authors also show that, despite doing less than mothers, fathers too are doing huge amounts of childcare. Indeed, they find that both mothers and fathers are reporting 3½ more one‐hour time slots of childcare per day than estimates from the UK Time Use Survey suggested for 2015. This suggests that while the gender gap in childcare time has not closed, both parents are taking a large share of the additional childcare and education responsibilities they are confronted with. The newly acquired role of fathers may permanently change how families and employers view paternal responsibilities and lead to long‐term changes in attitudes towards gender and families in the work environment.

### 4. School closures and children's education

The environment for learning that parents can provide may make a long‐lasting difference in the education outcomes of children, and better‐off families tend to provide more of the resources that children need to develop and learn. The extended period of school closures that started on 23 March and that, for many children, is unlikely to be over during the current school year risks making these differences even more significant as families assume a much larger share of the responsibility for educating their children. Moreover, the equalising force that schools represent during regular term time, with the provision of broadly standardised curriculums and safe learning environments, may have been jeopardised by their physical closure and lack of direction on the type of virtual resources and support for parents and children that schools were meant to provide.

To start investigating these issues, Andrew et al. (2020b) draw on data from the same IFS/IoE study and describe how the children in primary and secondary schools are spending their days during lockdown and what resources they have available. The study finds that children in better‐off families are spending more time in almost every single educational activity than their peers from the worse‐off fifth of families, with the overall difference exceeding one hour per day. Moreover, the gaps in learning times across economic groups are more pronounced for activities that involve active engagement with school teachers and private tuition, which may all be especially productive. Private tuition is also more common for children of better‐off families, with only 9 per cent of children in the bottom fifth of families by family earnings having private tuition classes compared with close to 20 per cent in the top income group.

One key consideration concerns the resources that schools are providing to support children's learning activities. Andrew et al. (2020b) show that private schools and the state schools that children from better‐off families attend are providing more activities that involve active engagement between teachers and students. These include online classes, videochats and online chats, and are likely to be more productive than more passive online resources and home learning packs.

These gaps mean that children from better‐off families are not only doing a higher quantity of home learning, they also have access to potentially higher‐quality support from schools and home learning opportunities. In addition, the activities that children from better‐off families are doing may also require less parental support because they involve a more active engagement with teachers and tutors. This means that they are less likely to overload parents with additional demands and to depend on the ability and availability of parents to be completed. It is almost certain that these factors will coalesce to widen an already prominent attainment gap between children from poorer and better‐off backgrounds in England. Unless there is a concerted effort to help these children once schools reopen, these wider gaps may well become permanent.

### 5. Health risks

The health risks of COVID‐19 so far have also not been evenly spread. The symptoms of the virus are more likely to be severe, and more likely to be fatal, for men than for women, and much more likely to be so for the old than for the young.^17^ Because people with certain underlying health conditions are especially badly hit, there is also a socio‐economic gradient in medical vulnerability to the virus.

**FIGURE 10:**
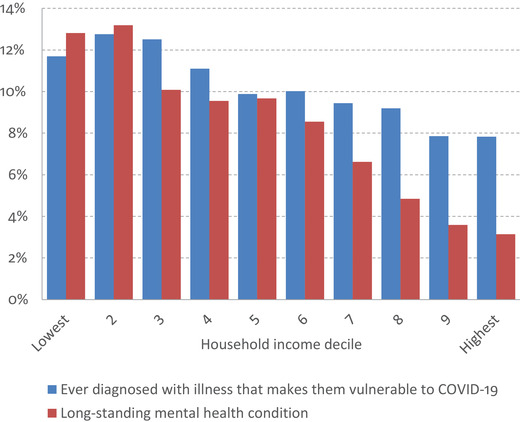
Medical vulnerability to COVID‐19 or the effects of social isolation, by income *Note*: Diagnoses include asthma, congestive heart failure, coronary heart disease, emphysema, chronic bronchitis, cancer or malignancy, diabetes and high blood pressure. Mental health based on self‐reported mental health condition lasting or expected to last over 12 months. Deciles based on equivalised net household incomes, using modified OECD equivalence scale. *Source*: UK Household Longitudinal Survey wave 9 (ever diagnosed) and Family Resources Survey 2018–19 (mental health).

Figure 10 shows that lower‐income individuals are more likely to have a health condition that makes them vulnerable to COVID‐19. Those in the bottom three deciles of the income distribution are around 50 per cent more likely to have been diagnosed with an illness that puts them at heightened medical risk than those in the top two deciles. The figure also shows that the prevalence of mental health conditions is far higher among lower‐income individuals: around 13 per cent in the bottom decile report having a long‐standing mental health condition, compared with just 3 per cent in the top decile. This means that those on lower incomes may also be more vulnerable to increased social isolation during the pandemic.

**FIGURE 11:**
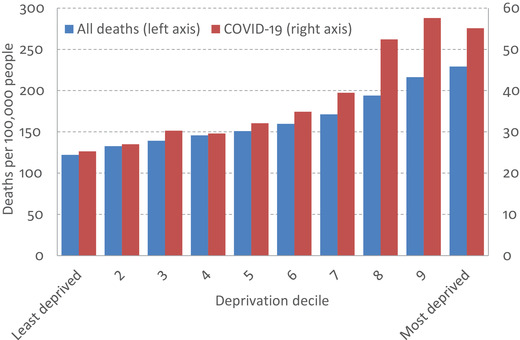
Age‐standardised death rates for deaths occurring between 1 March 2020 and 17 April 2020, by local area deprivation *Note*: Shows age‐standardised mortality rates, standardised to the 2013 European Standard Population. Deprivation deciles are defined using the ONS 2015 Index of Multiple Deprivation (IMD) series. *Source*: Office for National Statistics (ONS) mortality statistics (https://www.ons.gov.uk/peoplepopulationandcommunity/birthsdeathsandmarriages/deaths/bulletins/deathsinvolvingcovid19bylocalareasanddeprivation/deathsoccurringbetween1marchand17april).

There has been a steep socio‐economic gradient in deaths from COVID‐19, as shown in Figure 11. Between the start of March and the middle of April, age‐adjusted death rates in the most deprived tenth of areas in the UK were more than double those in the least deprived tenth of areas, measured by the Index of Multiple Deprivation (IMD). Death rates are generally higher in more deprived areas, but the gradient in COVID‐19 deaths has been considerably steeper than the gradient in total deaths (both when looking at total deaths during the COVID‐19 pandemic, as shown in the figure, and when looking at total deaths in previous years), especially at the top (most deprived part) of the distribution. This is likely to reflect a combination of heightened vulnerability to the virus if you get it, and higher exposure to getting it in the first place: underlying health conditions that put more deprived people at higher medical risk to the virus, as well as differences in occupations and working conditions (as discussed above), modes of transport and living environment that increase their risk of infection.

The knock‐on effects of COVID‐19 on the rest of the healthcare system may also disproportionately affect more deprived individuals. The NHS has moved to free up hospital beds to cope with increased demand during the pandemic, partly through the postponement of non‐urgent elective operations. Emergency care has also been affected: NHS England (2020) reports, for April, the lowest number of total accident and emergency (A&E) attendances since records began, 57 per cent lower than the same month last year. The number of emergency admissions was 39 per cent lower than in April 2019.

Propper, Stoye and Zaranko (2020, this issue) show that whilst elective hospital admissions are evenly spread across more and less deprived areas, emergency admissions are concentrated in more deprived areas. In 2017, the number of emergency admissions in the highest IMD decile was 70 per cent higher than in the lowest decile (note that because around a tenth of the population live in each IMD decile, this can roughly be interpreted as the difference in rates). To the extent that the decline in emergency attendances and admissions reflects lower demand due to self‐isolation or a reduction in workplace accidents, this may not be a bad thing. But if it reflects people in need of medical treatment staying away from hospitals in fear of catching the virus – and therefore storing up health problems for the future – this may further exacerbate health inequalities (and pressure on health services) in the future.

## IV. Implications for future inequalities

As we have seen, the COVID‐19 health crisis and the resulting lockdown have interacted with existing divides by income, age, gender and ethnicity, exacerbating many existing inequalities and opening up new fissures – such as between those whose jobs can and cannot be done from home – which are themselves often correlated with existing inequalities (for example, by income). Younger workers and those on low incomes are much more likely to have lost their job and experienced a reduction in earnings during the lockdown. The self‐employed and workers with less secure work arrangements have also been more likely to report negative impacts. Key workers, who often face more health risks, are more likely to be lower paid, female and from some ethnic minority groups. In general, health impacts have been unequal, with higher death rates among certain occupations, ethnic minority groups and poorer localities. Children in poorer families have lost more from school closures, and those who would have entered work this year face the potential for long‐term scarring from the collapsing labour market. In contrast, individuals with higher levels of education and higher earnings are more likely to be able to carry out their work activities from their home, to have space at home to educate their children and to have savings to cover any unforeseen expenditures.

Not all of the immediate impacts of the crisis point to increases in inequality. For example, the pension and housing wealth of richer groups may be taking a big hit, at least for now, as stock and house prices fall; and the cap on furlough payments and the very low level of out‐of‐work benefits relative to earnings mean that some higher earners will see a very large fall in incomes. But there are many factors that point the other way, and over the longer term we might worry more about the futures of those who have lost their jobs entirely or are at risk of doing so as their sectors face persistent struggles (which is much more likely among low earners), than about the smaller number of higher earners who are currently furloughed.

As we emerge from the pandemic, how can we prevent it from deepening existing inequalities and creating new fissures in society?

Consider three key pre‐COVID challenges: (i) ‘levelling up’ prosperity across the UK; (ii) generational inequalities; and (iii) gender inequality. In each case, the pandemic might have moved us backwards – at least initially. In terms of levelling up, some of the most deprived areas are also those where the most vulnerable live and where new jobs will be slowest to appear. They too are the places where high streets, often thought to be central to a thriving community, are most fragile and are now further threatened by the boost to online commerce. In terms of generational inequalities, younger generations are likely to face the largest scarring effects on job prospects and educational attainment, paying a larger amount of the cost of lockdown through higher taxes in both the short and longer terms. Moreover, any slowdown in the move away from fossil fuels, caused perhaps by a fall in oil prices, could reduce the adoption of green technology, saddling younger generations with a poorer environment. In terms of gender inequalities, unlike previous recessions this does not look like one in which the economic impacts will fall mostly on men. Although more likely to be in jobs that can be done from home, and more likely to be key workers, women are more likely to work in shut‐down sectors and, crucially, have been taking on most of the additional childcare required by school and nursery closures. For the least‐educated mothers, the impact looks particularly severe.

That said, closer inspection suggests there is scope for some aspects of the crisis to be turned into opportunities. Society may have an opportunity not only to address some of the most adverse impacts of the pandemic, but also to tackle some of the most pressing underlying longer‐term inequalities.

The pandemic has focused attention on the multiple sources of deprivation in certain areas of the UK. Health, economic and educational disadvantage come together in pockets of severe deprivation and vulnerability. This should put additional urgency on the need for effective place‐based policies that address levelling‐up and build resilience in these communities.

The experiences of people working from home during the pandemic, and the new technologies that have become in common usage, could provide the tipping point for a change in the way we work. There would, as mentioned above, be a risk that any benefits of that largely bypass lower‐income groups because the kinds of roles that they have tend to be less amenable to remote working. Successful education and training policy may be needed to counter that risk. On the other hand, an increase in the prevalence and/or productivity of remote working could be particularly important for women, who we know are often constrained during motherhood by seeking work close to their home. If ways of working do change, cities such as London may become less attractive and may suck up fewer of the highly educated and highly skilled workers, which might in itself be seen as an opportunity to spread prosperity more evenly across the country. There would again be dangers to be navigated here though: we want levelling‐up, not levelling‐down, and there are agglomeration benefits of large and successful cities.

For younger generations, there is the immediate challenge of repairing lost education investments and the likely longer‐term impacts of graduating during the lockdown. These losses will probably be most serious among lower‐income families and those with lower educational qualifications.^18^ The pandemic has highlighted educational inequalities. There is an opportunity here to rethink further education and vocational training to make us more resilient to big shocks that change the returns to different skills after people have entered the labour market, to focus on the skills that will be needed in an IT‐driven economy, and in a way that can provide the widespread human capital that will complement new technologies needed to achieve the net‐zero sustainable growth.

We have seen that women have been particularly adversely impacted by the enhanced dual pressures from childcare and work. But we have also documented an increase in childcare by men, even though it has not reduced the inequality in childcare time between men and women. The enhanced experiences of childcare among men over this period could, in the longer term, help to provide some of the change in social norms necessary to provide an even balance in childcare that has been so hard to achieve to date. Given the key role of childcare time, and time spent away from employment while looking after children, in driving the gender wage gap,^19^ that could be an important change. Women are also more likely to be key workers, and any pressure to increase the pay of key workers could help reduce gender inequalities, as well as income inequality overall.

There are other important legacies that the pandemic could have for the underlying structure of the economy, and some of these would be further challenges for inequality. One key example may relate to firms. The pandemic has refocused attention on the role of firms as places where good matches between workers and employers can provide enhanced productivity, giving better wages and some protection against adverse shocks. The Coronavirus Job Retention Scheme for furloughed workers was, in large part, an attempt to preserve those valuable employer–employee links. However, firms can also exploit their power in the product market and in the labour market in ways that are detrimental to consumers and also to workers. Large, already‐successful firms could find themselves in a position to capture a larger market share and have greater influence on wage‐setting as a result of this crisis, with smaller competitors – or potential competitors – unable to weather the temporary collapse in demand that they are facing or to adapt as radically as they might need to in order for working practices to be compatible with social distancing. The recent provisional backing of the UK's competition watchdog of Amazon's increased stake in Deliveroo is a case in point. There had already been concerns raised in the pre‐COVID environment that certain large firms have become ‘too’ powerful. One important line of research has been arguing that this has become increasingly the case in the last two or three decades across a wide range of economies, with an unjustifiable rise in market shares and a fall in labour share.^20^ The post‐pandemic world could provide an opportunity to rethink competition policy and re‐examine the balance of power between firms and workers in wage‐setting. This is not straightforward to police: price rises may simply reflect equilibrium responses to shifts in demand and supply, rather than changes in market power. We will need to be on the front foot with this: consolidations and anti‐competitive practices could happen quickly and are much harder to deal with after the event.

The scale of the furlough scheme and the support for the self‐employed during the lockdown, together with increases in the generosity of the existing safety net, have been key policy responses to the pandemic in the UK. These large‐scale interventions, and the experience of such a seismic shock that households were not prepared for, could change attitudes towards the role of government support, bringing about a new focus on social insurance.

One area where we might hope to see a shift in attitudes is in relation to ethnic divides, which have been highlighted by the pandemic both through exposure to infection and health risks, including mortality, and through exposure to loss of income. The much larger adverse health impacts on ethnic groups relative to the white British population cannot be explained simply by their age and location. While many minority groups live disproportionately in areas such as London and Birmingham, which have more COVID‐19 deaths, most minorities are also younger on average than the population as a whole, which should make them less vulnerable. The impact on different ethnic groups is a result of a complex set of economic, social and health‐related factors. A desire to understand and address these adverse impacts, together with the evident importance of the ethnic minority population as key workers, could well change attitudes and reverse some of the long‐standing economic and social inequalities that affect ethnic minorities.

In summary, it is clear from the evidence presented here that the COVID‐19 pandemic has exacerbated existing inequalities and brought to the fore other inequalities that were perhaps less of a concern prior to the pandemic. Without a well‐thought‐out policy response, the post‐COVID world could see inequalities worsening further. There are opportunities too though – not least because with more people having experienced state support, more people working at home, more men engaged in childcare and more focus on ethnic and educational inequalities, there could be important changes of attitudes. These could alter the political economy which, in the end, underlies all policymaking, perhaps fostering a desire to establish a more resilient economy that generates a fairer system of rewards.

The need to be on the front foot is paramount, however. The crisis has had many widely varying immediate impacts and much policy attention has rightly been consumed by those, but we will have to be thinking about the medium‐ and longer‐term legacies very soon if we are to respond effectively. Addressing the consequences of substantial career disruption after it has happened, or addressing a lack of competition after market consolidation has happened, is very difficult – the role of pre‐emptive and forward‐looking policy will be crucial. Not only that, but the deep challenges we already had coming into this crisis will, for the most part, remain or be magnified, and we must not forget about them.

## Footnotes

1 Costa Dias, Joyce and Parodi, 2018.

2 Department for Work and Pensions, 2020.

3 Cribb, Hood and Joyce, 2016.

4 Joyce and Xu, 2019.

5 Gibbons, Overman and Pelkonen, 2013; Overman, 2019.

6 Bennett et al., 2018.

7 Joyce and Xu, 2019.

8 Hood, 2017.

9 See Adam, Miller and Waters (2020).

10 Platt and Warwick, 2020, this issue.

11 Benzeval et al., 2020.

12 Office for National Statistics, 2020a.

13 Office for National Statistics, 2020b.

14 Platt and Warwick, 2020, this issue.

15 Hupkau and Petrongolo, 2020; Alon et al., 2020.

16 Benzeval et al., 2020.

17 Office for National Statistics, 2020c.

18 For example, see Cribb, Hood and Joyce (2017).

19 Costa Dias, Joyce and Parodi, 2018.

20 De Loecker, Eeckhout and Unger, 2020.
